# Trends in Patient-Reported Outcomes Measurement Information System (PROMIS) Utilization in Orthopedic Sports Medicine: A Scoping Review

**DOI:** 10.31486/toj.23.0054

**Published:** 2023

**Authors:** Nicholas A. Ott, Colby L. Barber, Steve E. Jordan

**Affiliations:** ^1^ Orthopedic Sports Medicine, Andrews Research and Education Foundation, Gulf Breeze, FL; ^2^ Department of Orthopedics, Andrews Institute, Gulf Breeze, FL

**Keywords:** *Patient reported outcome measures*, *review*, *sports medicine*

## Abstract

**Background:** The Patient-Reported Outcomes Measurement Information System (PROMIS) developed by the National Institutes of Health provides a standardized method for collecting outcomes data from sports medicine patient populations.

**Methods:** The objective of this scoping review is to report on PROMIS utilization in orthopedic sports medicine research and practice. We searched PubMed, ScienceDirect, and Cochrane Library using keywords and database-specific subject headings to identify studies that reported PROMIS utilization. Inclusion criteria were the use or mention of PROMIS in any population of patients commonly treated by orthopedic sports medicine physicians.

**Results:** Following a screening process, we included 67 studies published from 2019 through 2022 in this review. A near-equal distribution of studies was published per year during this period. Twenty-four domains were assessed across the studies. Among studies with adult populations, the Pain Interference (45 studies, 67%) and Physical Function (37 studies, 55%) domains were the 2 most reported by researchers. Upper Extremity (4 studies, 6%) and Mobility (3 studies, 4%) were the 2 most used domains in studies involving pediatric populations.

**Conclusion:** Our results demonstrate consistent utilization of PROMIS domains in the field of sports medicine. Researchers commonly used PROMIS with other legacy measures, comparing the scores to one another to assess responsiveness and validity. This review provides evidence that PROMIS domains are being used to collect data on a variety of factors related to sports medicine patient outcomes that may help physicians better understand the complexities of the recovery and rehabilitation process.

## INTRODUCTION

In the field of orthopedics, patient-reported outcome measures (PROMs) are commonly used because of the transition from volume-based to value-based health care and the increased focus on outcomes rather than costs.^1^ Historically, numerous legacy PROMs have been used to collect information from specific subsets of orthopedic patients. The International Knee Documentation Committee Subjective Knee Evaluation Form, Lysholm Knee Scoring Scale, and Tegner Activity Scale are 3 of the most common forms used to evaluate patient-reported outcomes in patients who undergo anterior cruciate ligament (ACL) reconstruction surgery.^2^ One limitation of these legacy PROMs is that many forms are often used to track outcomes. In a review of 119 ACL reconstruction studies published in high impact factor orthopedic journals, Makhni et al reported that 16 different PROMs were used to assess outcomes.^2^ This variability creates challenges when comparing measures from different populations and attempting to draw evidence-based conclusions.

The National Institutes of Health developed the Patient-Reported Outcomes Measurement Information System (PROMIS) to provide a standardized method of recording PROMs across common medical conditions.^3^ Domains within the system include questions related to physical function, fatigue, and emotional distress, among others, and can be adapted for a variety of health conditions or focuses.^3-5^ PROMIS can be administered as individual short forms or via computerized adaptive testing (CAT). CAT has demonstrated improved measurement precision when compared to fixed-format administration.^3,6-8^ The improved precision of CAT theoretically allows for obtaining the same or better-quality data while reducing the survey burden on the patient.^9^

The efficacy of PROMIS in orthopedics is well studied, and the system has demonstrated responsiveness and feasibility.^10-12^ In patients with orthopedic injuries or conditions, Physical Function is the most commonly studied domain^13^ and has been assessed in patient populations with musculoskeletal conditions of the foot and ankle, upper and lower extremities, and spine.^13-15^

An up-to-date assessment of PROMIS use in orthopedic sports medicine research and practice is warranted.^16^ The objective of this scoping review is to report on the use of PROMIS in the field of sports medicine.

## METHODS

### Review Design

We followed the Preferred Reporting Items for Systematic Reviews and Meta-Analyses (PRISMA) guidelines when designing the protocol for this scoping review^17^ and consulted a research librarian who reviewed and approved our search methods and strategy.

### Databases and Search Strategy

We conducted a comprehensive search of PubMed, ScienceDirect, and Cochrane Library using keywords and database-specific subject headings to identify studies that reported the use of PROMIS domains in sports medicine patient populations from January 1, 2019, to October 18, 2022 (Appendix). Search results were limited to research articles and case series. Case reports, systematic reviews, editorials, letters to the editor, and studies not written in English were eliminated either before or during the screening process.

### Inclusion and Exclusion Criteria

Inclusion criteria included the use of PROMIS domains for patients treated by orthopedic sports medicine physicians. Studies involving both pediatric and adult populations were included in our review. Exclusion criteria were nonorthopedic or non-sports medicine–related patient populations or interventions. Studies were included if they used at least 1 PROMIS domain, had an experimental or observational design, and were peer-reviewed.

### Study Selection

All titles and abstracts from the database searches were imported into Covidence (Veritas Health Innovation Ltd), an online systematic review management software.^18^ The article selection process had 2 steps. First, 2 authors (NAO and CLB) independently reviewed the titles and abstracts of the imported articles using the predefined inclusion and exclusion criteria. Articles that either 1 or both authors determined to be applicable were moved to full-text review. In the second step, the same 2 authors determined article eligibility through independent review of the full-text articles. In the event of a conflict, the 2 authors discussed and reached a verdict on whether to include or exclude the article.

### Data Analysis

All studies included in the review were evaluated between October 2022 and December 2022 and were organized according to publication year, population, study design, and sample size (Table). We conducted a PROMIS-specific analysis to capture the various domains reported in the studies, as well as the administration methods. Qualitative analysis of the studies included assigning tags to track information related to the location of the intervention (eg, upper extremity, lower extremity, knee) and to themes such as the minimal clinically important difference (MCID), comparison of legacy outcome measures to PROMIS, PROMIS validation and responsiveness, and participant demographics and psychosocial characteristics.

**Table.** Characteristics of the Studies Included in the Review

Study	Population	Study Design	N
Beletsky et al, 2019^22^	Upper extremity patients	Retrospective cohort study	122
Blanchett et al, 2019^24^	Upper and lower extremity patients	Cohort study (prospective observational study)	833
Cheng et al, 2019^29^	Upper extremity patients	Retrospective cohort study	179
Fidai et al, 2019^36^	Lower extremity patients	Retrospective cohort study	527
Fu et al, 2019^41^	Upper extremity patients	Retrospective cohort study	179
Gulledge et al, 2019^43^	Upper and lower extremity patients	Cohort study (prospective observational study)	2,952
Kahan et al, 2019^48^	Upper extremity patients	Cohort study (prospective observational study)	62
Lizzio et al, 2019^10^	Upper and lower extremity patients	Cross-sectional study	581
Makhni et al, 2019^54^	Upper and lower extremity patients	Cross-sectional study	236
Meldau et al, 2019^55^	Upper extremity patients	Cross-sectional study	172
Simon et al, 2019^73^	Lower extremity patients	Cross-sectional study	69
Sochacki et al, 2019^74^	Orthopedic surgeons	Case series	21
Tyser et al, 2019^78^	Upper extremity patients	Cohort study (prospective observational study)	825
Wikstrom and Song, 2019^79^	Lower extremity patients	Cross-sectional study	90
Wright et al, 2019^80^	Upper and lower extremity patients	Cross-sectional study	7,500
Beletsky et al, 2020^21^	Upper extremity patients	Cohort study (prospective observational study)	190
Bernstein et al, 2020^23^	Upper extremity patients	Cohort study (prospective observational study)	188
Collis et al, 2020^32^	Lower extremity patients	Retrospective cohort study	260
Friedman et al, 2020^40^	Lower extremity patients	Case series	11
Kuhns et al, 2020^50^	Lower extremity patients	Cohort study (prospective observational study)	113
Lu et al, 2020^52^	Upper extremity patients	Cohort study (prospective observational study)	175
Lu et al, 2020^53^	Lower extremity patients	Case series	152
Nadarajah et al, 2020^59^	Upper extremity patients	Cross-sectional study	195
Nwachukwu et al, 2020^61^	Lower extremity patients	Case series	250
Nwachukwu et al, 2020^62^	Lower extremity patients	Case series	96
Okoroha et al, 2020^63^	Lower extremity patients	Retrospective cohort study	73
Okoroha et al, 2020^64^	Lower extremity patients	Randomized controlled trial	18
Paget et al, 2020^65^	Lower extremity patients	Cross-sectional study	553
Roberts and Voloshin, 2020^68^	Upper extremity patients	Retrospective cohort study	2,632
Schafer et al, 2020^69^	Lower extremity patients	Cohort study (prospective observational study)	100
Schreiner et al, 2020^71^	Lower extremity patients	Cohort study (prospective observational study)	27
Shamrock et al, 2020^72^	Lower extremity patients	Retrospective cohort study	275
Andrews et al, 2021^19^	Lower extremity patients	Retrospective cohort study	173
Bodendorfer et al, 2021^25^	Lower extremity patients	Cohort study (prospective observational study)	124
Broughton et al, 2021^26^	Upper extremity patients	Retrospective cohort study	57
Browning et al, 2021^27^	Lower extremity patients	Retrospective cohort study	2,170
Day et al, 2021^34^	Lower extremity patients	Cohort study (prospective observational study)	1,126
Forlenza et al, 2021^37^	Upper extremity patients	Cohort study (prospective observational study)	112
Franovic et al, 2021^38^	Lower extremity patients	Retrospective cohort study	166
Franovic et al, 2021^39^	Upper extremity patients	Cross-sectional study	196
Gulledge et al, 2021^11^	Lower extremity patients	Cohort study (prospective observational study)	100
Guo et al, 2021^44^	Upper extremity patients	Cross-sectional study	413
Hartwell et al, 2021^45^	Lower extremity patients	Retrospective cohort study	86
Jildeh et al, 2021^47^	Upper and lower extremity patients	Case series	177
Minaie et al, 2021^57^	Uninjured collegiate athletes	Cross-sectional study	194
Moran et al, 2021^58^	Lower extremity patients	Case series	42
Patel et al, 2021^66^	Upper extremity patients	Retrospective cohort study	39
Tyser et al, 2021^77^	Upper extremity patients	Cohort study (prospective observational study)	268
Yagnik et al, 2021^81^	Upper extremity patients	Cohort study (prospective observational study)	127
Yedulla et al, 2021^82^	Lower extremity patients	Case series	137
Barnes et al, 2022^20^	Upper and lower extremity patients	Cohort study (prospective observational study)	188
Cheah et al, 2022^28^	Lower extremity patients	Retrospective cohort study	459
Christino et al, 2022^30^	Upper and lower extremity patients	Cross-sectional study	107
Colasanti et al, 2022^31^	Lower extremity patients	Cohort study (prospective observational study)	31
Cross et al, 2022^33^	Upper and lower extremity patients	Cohort study (prospective observational study)	153
Day et al, 2022^35^	Lower extremity patients	Cohort study (prospective observational study)	660
Gambhir et al, 2022^42^	Upper extremity patients	Case series	44
Hogan et al, 2022^46^	Lower extremity patients	Retrospective cohort study	119
Khalil et al, 2022^49^	Lower extremity patients	Cohort study (prospective observational study)	43
Lavoie-Gagne et al, 2022^51^	Upper extremity patients	Retrospective cohort study	107
Meta et al, 2022^56^	Upper extremity patients	Retrospective cohort study	184
Nicholson et al, 2022^60^	Upper extremity patients	Case series	193
Pietroski et al, 2022^67^	Upper extremity patients	Retrospective cohort study	180
Schaffer et al, 2022^70^	Lower extremity patients	Cohort study (prospective observational study)	95
Kubala et al, 2022^75^	Upper and lower extremity patients	Cross-sectional study	149
Tramer et al, 2022^76^	Lower extremity patients	Randomized controlled trial	45
Ziedas et al, 2022^83^	Upper extremity patients	Retrospective cohort study	338

## RESULTS

**Figure 1.**
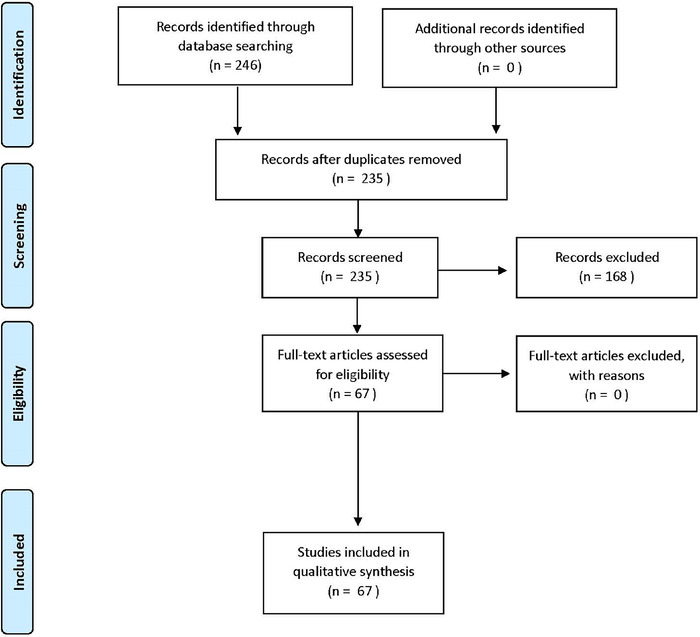
Preferred Reporting Items for Systematic Reviews and Meta-Analyses (PRISMA) literature flow diagram.^17^

The preliminary search yielded 246 publications. Following duplicate removal, we reviewed 235 publications by titles and abstracts. After resolution of conflicts related to article inclusion or exclusion, an agreement rate >80% was calculated between the authors. Following this step, 67 publications were moved to the full-text review stage, and all 67 were determined to be eligible for inclusion in the review^10,11,19-83^ (Figure 1).

### Study Publication Date and Design

A near-equal distribution of studies was published per year: 2019 (15 studies, 22%), 2020 (17, 25%), 2021 (18, 27%), and 2022 (17, 25%). Study designs included cohort study or prospective observational study (22, 33%), retrospective cohort study (20, 30%), cross-sectional study (13, 19%), case series (10, 15%), and randomized controlled trial (2, 3%).

### Patient Population

Included publications reported PROMIS utilization in a variety of orthopedic sports medicine patient populations. PROMIS was more commonly utilized in sports medicine patient populations with conditions affecting the lower extremity (31, 46%) vs the upper extremity (24, 36%). Ten studies (15%) included patients with upper and lower extremity conditions. The studies included analyses of joint-specific populations related to the knee (19, 28%), shoulder (19, 28%), hip (8, 12%), ankle (5, 7%), and elbow (5, 7%).

### PROMIS Domains and Administration Methods

**Figure 2.**
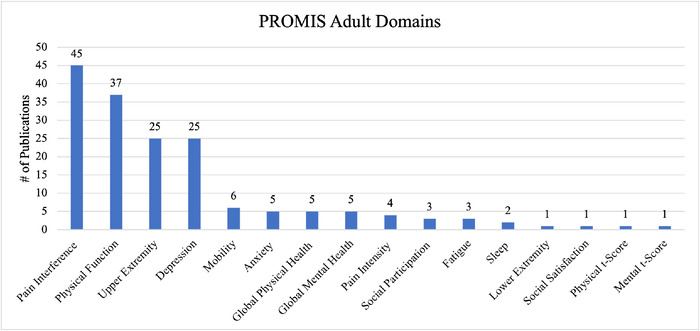
**Patient-Reported Outcomes Measurement Information System** (**PROMIS) adult domains and frequency of reporting in studies included in this review.**

Sixteen PROMIS domains were reported in publications involving adult populations: Pain Interference (45, 67%), Physical Function (37, 55%), Upper Extremity (25, 37%), Depression (25, 37%), Mobility (6, 9%), Anxiety (5, 7%), Global Physical Health (5, 7%), Global Mental Health (5, 7%), Pain Intensity (4, 6%), Social Participation (3, 4%), Fatigue (3, 4%), Sleep (2, 3%), Lower Extremity (1, 1%), Social Satisfaction (1, 1%), Physical t-Score (1, 1%), and Mental t-Score (1, 1%) (Figure 2).

**Figure 3.**
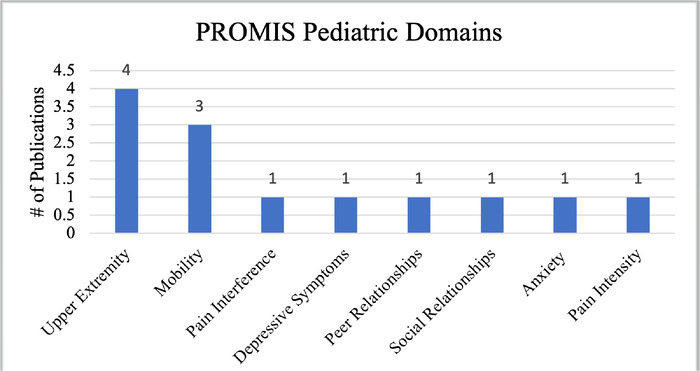
**Patient-Reported Outcomes Measurement Information System** (**PROMIS) pediatric domains and frequency of reporting in studies included in this review.**

Studies involving pediatric populations utilized 8 pediatric-specific domains: Upper Extremity (4, 6%), Mobility (3, 4%), Pain Interference (1, 1%), Depressive Symptoms (1, 1%), Peer Relationships (1, 1%), Social Relationships (1, 1%), Anxiety (1, 1%), and Pain Intensity (1, 1%) (Figure 3).

In the 57 studies that identified a PROMIS administration method, CAT was the most common (48/57, 84%), with short forms used less often than CAT (9/57, 16%).

PROMIS domains were used as the only outcomes measure in approximately one-third of the studies (23, 34%).

### Legacy Patient-Reported Outcome Measures

Legacy PROMs were commonly reported alongside PROMIS domains (44, 66%). The specific legacy PROM used in each publication was largely dependent on the study population. For example, in publications focused on patients undergoing shoulder surgery, such as total shoulder arthroplasty, the American Shoulder and Elbow Surgeons score was commonly reported (10, 15%).

### Qualitative Themes

The authors developed and assigned tags to identify a variety of themes in the included publications: comparison of PROMs to PROMIS domains (19, 28%), impact of patient characteristics (eg, workers’ compensation) on PROMIS scores (14, 21%), MCID (12, 18%), role of psychosocial elements (eg, mental health and tobacco use) on PROMIS scores (13, 19%), administration method and time constraints (11, 16%), coronavirus disease 2019 pandemic (1, 1%), and the application of PROMs to other languages (1, 1%).

## DISCUSSION

Our review of the literature from 2019 to 2022 identified publications reporting PROMIS use specifically in sports medicine populations and an increase in the volume of annual publications compared to nearly a decade ago.^13^ This increase is most likely multifactorial, and, as Horn et al noted, likely attributable to advances in administration methods (ie, CAT) and improved integration into electronic health record systems.^16^ PROMIS utilization in the field of orthopedic sports medicine follows similar patterns of use in the broader field of orthopedics, with PROMIS domains commonly used in parallel with legacy PROMs.^16^ Although several of the publications included in this review used PROMIS domains as the primary or only outcome measure (34%), most studies reported multiple legacy PROMs in conjunction with PROMIS domains (66%). Horn et al surmised that this phenomenon may be because of “familiarity with traditional measures, participation in registries that do not have PROMIS measures as part of the core set of measures, or a perceived lack of applicability in their patient populations.”^16^ Studies investigating the floor and ceiling effects of PROMIS domains and studies comparing the responsiveness of PROMIS domains to that of legacy PROMs represent natural steps to further validate PROMIS domains as capable of capturing meaningful outcome measures.^37,43,59,72^

The breadth of PROMIS domains used in the publications included in this review highlight the fact that PROMIS is capable of excellent generalizability. Bernstein et al investigated the correlation between adult and pediatric PROMIS domains in pediatric sports medicine patients.^23^ They concluded that the adult PROMIS domains could be used for both adult and pediatric patients in an orthopedic sports medicine clinic because of the high degree of correlation between the different versions of the domains.^23^ Additionally, researchers have investigated the validity of a Spanish version of certain PROMIS domains, mainly Physical Function.^81^ While further investigation is needed, these studies demonstrate that PROMIS is successfully being applied to diverse sports medicine patient populations.

Domains such as Sleep allow PROMIS to be tailored to investigate the multifactorial recovery process following orthopedic surgeries. Cheah et al analyzed the association between sleep disturbance and femoroacetabular impingement syndrome.^28^ Sochacki et al measured orthopedic surgeon burnout through a combination of the Maslach Burnout Inventory, PROMIS domains, and a wearable device.^74^ Sochacki et al used PROMIS-29 that includes the following domains: Physical Function, Anxiety, Depression, Fatigue, Sleep, Social Participation, Pain Interference, and Pain Intensity.^74^ PROMIS is capable of capturing outcomes beyond Physical Function to aid researchers in better understanding the complex, multifactorial conditions that affect orthopedic sports medicine surgeons and patients.

Only Lizzio et al discussed the feasibility of PROMIS CAT administration in a sports medicine clinic.^10^ However, our experience has highlighted the importance of having a streamlined administration and data collection system to capture patient survey responses. To incorporate PROMIS CAT domains into our sports medicine research we have used PatientIQ (PatientIQ) and REDCap (Vanderbilt University). PatientIQ is a third-party subscription-based platform that securely manages patient-reported outcomes data and can administer PROMIS CAT surveys to designated patients. REDCap is a web-based secure data collection instrument that also has the capability of administering PROMIS CAT surveys. The REDCap tool is managed in house by research team members. Both platforms can be used to administer other legacy outcome measures either individually or alongside the PROMIS domains. Future investigation into the most widely used platforms for PROMIS CAT administration may provide valuable insight for orthopedic sports medicine practices interested in collecting PROMIS data for research and clinical outcomes data.

### Limitations

Limitations of our scoping review are that we did not conduct an analysis of the quality of the included studies and did not perform a risk assessment of bias.

## CONCLUSION

Our results demonstrate a consistent utilization of PROMIS domains in sports medicine clinical research and practice. Pain Interference and Physical Function were the 2 most reported PROMIS domains in the included publications. While nearly one-third of the articles we reviewed used PROMIS as the only or primary outcome measure, researchers more commonly used PROMIS in addition to other legacy measures, often comparing the scores to assess responsiveness and validity. The development of third-party platforms such as PatientIQ that streamline administration and data capture procedures offers the potential for increased utility of PROMIS and its ability to provide data on a variety of factors contributing to patient outcomes.

## Notes

*This article meets the Accreditation Council for Graduate Medical Education and the American Board of Medical Specialties Maintenance of Certification competencies for Patient Care and Medical Knowledge.*

## Appendix. Search Strategies for Trends in Patient-Reported Outcomes Measurement Information System (PROMIS) Utilization in Orthopedic Sports Medicine: A Scoping Review

**Table:** Database: PubMed from 2019 to 2022

Set	Search Terms	Results
1	("Orthopedic Procedures"[Mesh] OR "Orthopedics"[Mesh] OR orthopedic[tiab] OR orthopaedic[tiab] OR orthopedics[tiab] OR orthopaedics[tiab] OR musculoskeletal[tiab] OR "neck"[MeSH Terms] OR neck[tiab] OR "spine"[MeSH Terms] OR spine[tiab] OR spinal[tiab] OR cervical[tiab] OR "lumbosacral region"[MeSH Terms] OR lumbosacral[tiab] OR lumbar[tiab] OR thoracic[tiab] OR "back"[MeSH Terms] OR back[tiab] OR "arthroplasty"[MeSH Terms] OR arthroplasty[tiab] OR "low back"[tiab] OR "lower back"[tiab] OR "shoulder"[MeSH Terms] OR shoulder[tiab] OR "elbow"[MeSH Terms] OR "elbow joint"[MeSH Terms] OR elbow[tiab] OR "hand"[MeSH Terms] OR hand[tiab] OR "hip"[MeSH Terms] OR hip[tiab] OR "knee"[MeSH Terms] OR "knee joint"[MeSH Terms] OR knee[tiab] OR "anterior cruciate ligament"[MeSH Terms] OR "anterior cruciate ligament"[tiab] OR trauma[tiab] OR "meniscus"[MeSH Terms] OR meniscus[tiab] OR "ankle"[MeSH Terms] OR "ankle joint"[MeSH Terms] OR ankle[tiab] OR "foot"[MeSH Terms] OR foot[tiab] OR "wrist"[MeSH Terms] OR "wrist joint"[MeSH Terms] OR wrist[tiab] OR "upper extremity"[MeSH Terms] OR "upper extremity"[tiab] OR "lower extremity"[MeSH Terms] OR "lower extremity"[tiab] OR "temporomandibular joint"[MeSH Terms] OR temporomandibular[tiab] OR "physiopathology"[Subheading] OR "Orthopedic Procedures"[Mesh] OR "Wounds and Injuries"[Mesh] OR "injuries"[Subheading] OR "Osteoarthritis"[Mesh] OR Osteoarthritis[tiab] OR "Arthritis"[Mesh] OR arthritis[tiab])	743,174
2	(PROMIS [tiab] OR "patient reported outcome measurement information system"[tiab] OR "patient reported outcomes measurement information system"[tiab])	2,493
3	#1 AND #2	1,269
4	#3 AND (“sports medicine” [tiab] OR “athletic” [tiab] OR “athlete” [tiab])	37

**Table:** Database: ScienceDirect via Elsevier from 2019 to 2022

Set	Search Terms	Results
1	(PROMIS OR “patient reported outcomes measurement information system”)	4,590
2	(“Sports medicine” OR “athlete” OR “athletic”)	29,974
3	#1 AND #2	288
4	#3 in review articles, research articles, and book chapters	193

**Table:** Database: Cochrane Library from January 2019 to October 2022

Set	Search Terms	Results
1	(Orthopedic OR orthopaedic OR orthopedics OR orthopaedics OR musculoskeletal OR neck OR spine OR spinal OR cervical OR lumbosacral OR lumbar OR thoracic OR back OR arthroplasty OR low back OR lower back OR shoulder OR elbow OR hand OR hip OR knee OR anterior cruciate ligament OR trauma OR meniscus OR ankle OR foot OR wrist OR upper extremity OR lower extremity OR temporomandibular OR physiopathology OR injury OR injuries OR Osteoarthritis OR arthritis)	156,556
2	(PROMIS OR "patient reported outcomes measurement information system")	1,121
3	#1 AND #2	512
4	#3 AND ("sports medicine" OR "athlete" OR "athletic") in Cochrane Reviews and Trials	16
